# (*Z*)-(1,3-Thia­zinan-2-ylideneamino)formo­nitrile

**DOI:** 10.1107/S1600536809008940

**Published:** 2009-03-19

**Authors:** Yu-wen Peng, Lin-hai Wu

**Affiliations:** a Jiang Nan University, Wu-Xi 214112, People’s Republic of China, and, Finance Department of Business School, Hunan Normal University, Changsha 412006, People’s Republic of China; b Jiang Nan University, Wu-Xi 214112, People’s Republic of China;

## Abstract

In the title mol­ecule, C_5_H_7_N_3_S, the thia­zine ring shows a conformation close to a half-boat. The Cremer & Pople puckering parameters of the thia­zine ring are *q*2 = 0.4645 (2) Å, θ = 132.4 (3) and ϕ = 285.52 (2)°. The packing is stabilized by inter­molecular N—H⋯N and C—H⋯S inter­actions.

## Related literature

For the crystal structures of thia­zine compounds, see: Kálmán, *et al.* (1977 ▶). For the biological activities of thia­zine-containing compounds, see: Soloway *et al.* (1978 ▶); Tomizawa *et al.* (1995 ▶). For bond-length data, see: Allen *et al.* (1987 ▶). For puckering parameters, see: Cremer & Pople (1975 ▶).
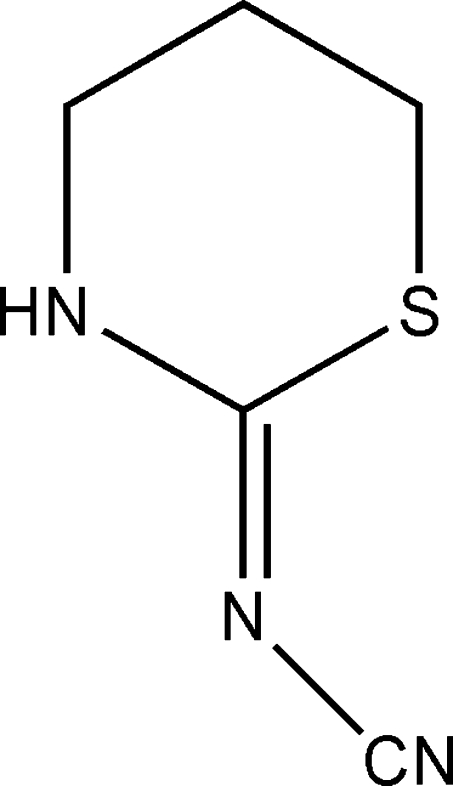


## Experimental

### 

#### Crystal data

C_5_H_7_N_3_S
*M* *_r_* = 141.21
Monoclinic, 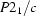

*a* = 7.0931 (14) Å
*b* = 12.689 (3) Å
*c* = 9.232 (3) Å
β = 128.617 (19)°
*V* = 649.2 (3) Å^3^
*Z* = 4
Mo *K*α radiation
μ = 0.40 mm^−1^
*T* = 153 K
0.42 × 0.11 × 0.06 mm

#### Data collection

Rigaku R-AXIS RAPID IP area-detector diffractometer
Absorption correction: multi-scan (*ABSCOR*; Higashi 1995 ▶) *T*_min_ = 0.849, *T*_max_ = 0.976
4731 measured reflections
1101 independent reflections
970 reflections with *I* > 2σ(*I*)
*R*_int_ = 0.032

#### Refinement

*R*[*F* ^2^ > 2σ(*F* ^2^)] = 0.066
*wR*(*F* ^2^) = 0.192
*S* = 1.09
1101 reflections
83 parameters
H-atom parameters constrained
Δρ_max_ = 1.14 e Å^−3^
Δρ_min_ = −0.32 e Å^−3^

### 

Data collection: *RAPID-AUTO* (Rigaku, 2004 ▶); cell refinement: *RAPID-AUTO*; data reduction: *RAPID-AUTO*; program(s) used to solve structure: *SHELXTL* (Sheldrick, 2008 ▶); program(s) used to refine structure: *SHELXTL*; molecular graphics: *SHELXTL*; software used to prepare material for publication: *SHELXTL*.

## Supplementary Material

Crystal structure: contains datablocks I, global. DOI: 10.1107/S1600536809008940/hg2486sup1.cif

Structure factors: contains datablocks I. DOI: 10.1107/S1600536809008940/hg2486Isup2.hkl

Additional supplementary materials:  crystallographic information; 3D view; checkCIF report

## Figures and Tables

**Table 1:** Hydrogen-bond geometry (Å, °)

*D*—H⋯*A*	*D*—H	H⋯*A*	*D*⋯*A*	*D*—H⋯*A*
N1—H1*A*⋯N3^i^	0.86	2.12	2.926 (4)	156
C3—H3*B*⋯S1^ii^	0.99	2.74	3.468 (3)	131

Symmetry codes: (i) 
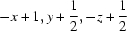
; (ii) 
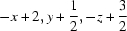
.

**Fig. 1.**
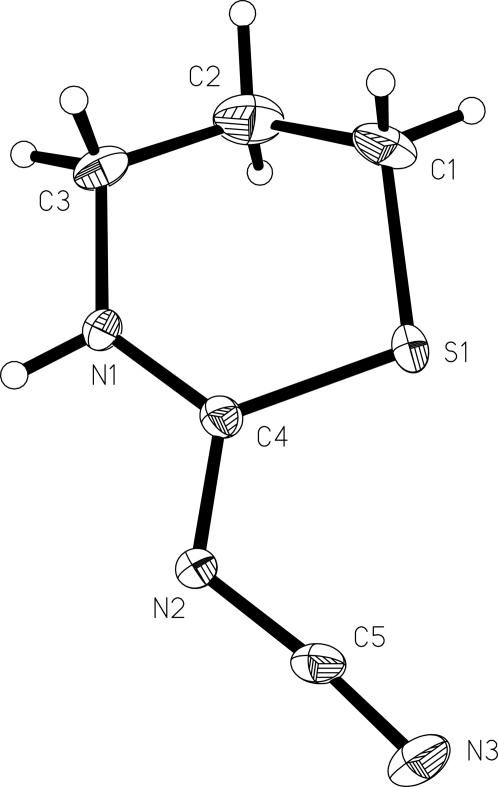
The molecular structure of (I), with atom labels and 40% probability displacement ellipsoids for non-H atoms.

## supplementary crystallographic information

### Comment

The frequent occurence of pesticide residues accidents and the introduction of Green Trade Barriers to protect human health threaten export and national agriculture market and highlights the importance of food safely.  Therefore, the development of pesticides with new chemical structures and high insecticidal activities with low residues is highly desirable. Consequently, spurred by the need for new pesticidal agents and the fact that many new effective pesticides possess heterocyclic rings in their structure, such as the thiazine ring (Soloway *et al.*, 1978; Tomizawa *et al.*, 1995), over the last few years, we have synthesized some novel thiazine derivatives. Here, we report the crystal structure of (*Z*)-(1,3-thiazinan-2-ylideneamino)formonitrile.

In (*Z*)-(1,3-thiazinan-2-ylideneamino)formonitrile (Fig. 1), all bond lengths are normal (Allen *et al.*, 1987) and in a good agreement with those reported previously (Kálmán, *et al.*, 1977). It is known that the imino tautomers can exist as two geometrical isomers, *syn* (*Z*) and anti (E), but in this crystal, only *Z* isomers have been observed. The thiazine ring shows a conformation near to half boat with the C2 atom deviating 0.618 (2) Å above the plane formed by S1, C1, N1, C3 and C4 [maximum least squares plane deviation for S1 0.053 (3) Å]. This geometry is proved by the puckering parameters q2 = 0.4645 (2) Å, θ = 132.4 (3)° and φ = 285.52 (2)° (Cremer & Pople, 1975). There are some weak N—H···N and C—H···S intermolecular interactions (see Table 1) which stabilize the title structure.

### Experimental

A mixture of dimethyl cyanocarbonimidodithioate 10 mmol (1.46 g) and 3-aminopropane-1-thiol (1.00 g, 11 mmol) was refluxed in absolute EtOH (20 ml) for 3 h. On cooling, the product crystallizes and was filtered and then recrystallized from absolute ethanol. Yield 1.20 g (85%). Single crystals suitable for X-ray measurements were obtained by recrystallization from ethanol at room temperature.

### Refinement

H atoms were positioned geometrically and refined using a riding model, with C—H = 0.99 Å, N—H = 0.86 Å with *U*_iso_(H) = 1.2 times *U*_eq_(C, N).

### Crystal data

**Table:**

C_5_H_7_N_3_S	*F*(000) = 296
*M**_r_* = 141.21	*D*_x_ = 1.445 Mg m^−^^3^
Monoclinic, *P*2_1_/*c*	Mo *K*α radiation, λ = 0.71073 Å
Hall symbol: -P 2ybc	Cell parameters from 2501 reflections
*a* = 7.0931 (14) Å	θ = 2.3–25.1°
*b* = 12.689 (3) Å	µ = 0.40 mm^−^^1^
*c* = 9.232 (3) Å	*T* = 153 K
β = 128.617 (19)°	Needle, colorless
*V* = 649.2 (3) Å^3^	0.42 × 0.11 × 0.06 mm
*Z* = 4	

### Data collection

**Table:**

Rigaku R-AXIS RAPID IP area-detector diffractometer	1101 independent reflections
Radiation source: Rotating Anode	970 reflections with *I* > 2σ(*I*)
graphite	*R*_int_ = 0.032
ω Oscillation scans	θ_max_ = 25.0°, θ_min_ = 3.2°
Absorption correction: multi-scan (*ABSCOR*; Higashi 1995)	*h* = −8→8
*T*_min_ = 0.849, *T*_max_ = 0.976	*k* = −14→15
4731 measured reflections	*l* = −10→10

### Refinement

**Table:**

Refinement on *F*^2^	Secondary atom site location: difference Fourier map
Least-squares matrix: full	Hydrogen site location: inferred from neighbouring sites
*R*[*F*^2^ > 2σ(*F*^2^)] = 0.066	H-atom parameters constrained
*wR*(*F*^2^) = 0.192	*w* = 1/[σ^2^(*F*_o_^2^) + (0.1515*P*)^2^ + 0.068*P*] where *P* = (*F*_o_^2^ + 2*F*_c_^2^)/3
*S* = 1.09	(Δ/σ)_max_ < 0.001
1101 reflections	Δρ_max_ = 1.14 e Å^−^^3^
83 parameters	Δρ_min_ = −0.31 e Å^−^^3^
0 restraints	Extinction correction: *SHELXTL* (Sheldrick, 2008), Fc^*^=kFc[1+0.001xFc^2^λ^3^/sin(2θ)]^-1/4^
Primary atom site location: structure-invariant direct methods	Extinction coefficient: 0.040 (14)

### Special details

**Table:**

Geometry. All e.s.d.'s (except the e.s.d. in the dihedral angle between two l.s. planes) are estimated using the full covariance matrix. The cell e.s.d.'s are taken into account individually in the estimation of e.s.d.'s in distances, angles and torsion angles; correlations between e.s.d.'s in cell parameters are only used when they are defined by crystal symmetry. An approximate (isotropic) treatment of cell e.s.d.'s is used for estimating e.s.d.'s involving l.s. planes.
Refinement. Refinement of *F*^2^ against ALL reflections. The weighted *R*-factor *wR* and goodness of fit *S* are based on *F*^2^, conventional *R*-factors *R* are based on *F*, with *F* set to zero for negative *F*^2^. The threshold expression of *F*^2^ > σ(*F*^2^) is used only for calculating *R*-factors(gt) *etc*. and is not relevant to the choice of reflections for refinement. *R*-factors based on *F*^2^ are statistically about twice as large as those based on *F*, and *R*- factors based on ALL data will be even larger.

### Fractional atomic coordinates and isotropic or equivalent isotropic displacement parameters (Å^2^)

**Table:**

	*x*	*y*	*z*	*U*_iso_*/*U*_eq_	
S1	0.97192 (13)	0.28689 (6)	0.64894 (10)	0.0263 (5)	
N1	0.7395 (4)	0.47140 (17)	0.5152 (3)	0.0213 (6)	
H1A	0.6246	0.5077	0.4223	0.026*	
N2	0.5695 (4)	0.33420 (19)	0.3151 (3)	0.0231 (7)	
N3	0.5490 (5)	0.1469 (2)	0.2340 (4)	0.0324 (8)	
C1	1.1467 (7)	0.3667 (3)	0.8561 (5)	0.0406 (10)	
H1B	1.0764	0.3613	0.9206	0.049*	
H1C	1.3136	0.3390	0.9404	0.049*	
C2	1.1539 (7)	0.4785 (3)	0.8161 (5)	0.0403 (9)	
H2B	1.2330	0.4840	0.7582	0.048*	
H2C	1.2538	0.5179	0.9345	0.048*	
C3	0.9066 (6)	0.5297 (3)	0.6889 (5)	0.0316 (8)	
H3A	0.8374	0.5342	0.7544	0.038*	
H3B	0.9245	0.6023	0.6600	0.038*	
C4	0.7430 (5)	0.3711 (2)	0.4833 (4)	0.0189 (7)	
C5	0.5676 (5)	0.2335 (2)	0.2802 (4)	0.0228 (7)	

### Atomic displacement parameters (Å^2^)

**Table:**

	*U*^11^	*U*^22^	*U*^33^	*U*^12^	*U*^13^	*U*^23^
S1	0.0269 (6)	0.0189 (6)	0.0199 (6)	0.0071 (3)	0.0081 (5)	0.0043 (2)
N1	0.0262 (13)	0.0119 (11)	0.0189 (13)	0.0008 (10)	0.0108 (12)	−0.0002 (9)
N2	0.0243 (13)	0.0159 (13)	0.0174 (13)	0.0017 (10)	0.0073 (11)	−0.0020 (9)
N3	0.0362 (17)	0.0226 (15)	0.0376 (18)	−0.0079 (12)	0.0226 (15)	−0.0118 (12)
C1	0.0317 (19)	0.052 (2)	0.0163 (17)	0.0024 (15)	0.0041 (15)	−0.0040 (14)
C2	0.040 (2)	0.044 (2)	0.032 (2)	−0.0136 (16)	0.0198 (18)	−0.0108 (15)
C3	0.0365 (19)	0.0224 (16)	0.0284 (18)	−0.0043 (14)	0.0165 (16)	−0.0111 (13)
C4	0.0206 (14)	0.0179 (14)	0.0165 (15)	0.0000 (11)	0.0107 (13)	0.0014 (11)
C5	0.0180 (14)	0.0265 (16)	0.0181 (15)	−0.0046 (12)	0.0085 (13)	−0.0056 (12)

### Geometric parameters (Å, °)

**Table:**

S1—C4	1.737 (3)	C1—C2	1.474 (6)
S1—C1	1.806 (4)	C1—H1B	0.9900
N1—C4	1.310 (4)	C1—H1C	0.9900
N1—C3	1.467 (4)	C2—C3	1.519 (5)
N1—H1A	0.8600	C2—H2B	0.9900
N2—C5	1.315 (4)	C2—H2C	0.9900
N2—C4	1.327 (4)	C3—H3A	0.9900
N3—C5	1.156 (4)	C3—H3B	0.9900
			
C4—S1—C1	103.52 (16)	C1—C2—H2C	108.8
C4—N1—C3	128.3 (3)	C3—C2—H2C	108.8
C4—N1—H1A	115.8	H2B—C2—H2C	107.7
C3—N1—H1A	115.9	N1—C3—C2	112.6 (3)
C5—N2—C4	119.3 (2)	N1—C3—H3A	109.1
C2—C1—S1	112.5 (3)	C2—C3—H3A	109.1
C2—C1—H1B	109.1	N1—C3—H3B	109.1
S1—C1—H1B	109.1	C2—C3—H3B	109.1
C2—C1—H1C	109.1	H3A—C3—H3B	107.8
S1—C1—H1C	109.1	N1—C4—N2	118.2 (3)
H1B—C1—H1C	107.8	N1—C4—S1	122.6 (2)
C1—C2—C3	113.7 (3)	N2—C4—S1	119.2 (2)
C1—C2—H2B	108.8	N3—C5—N2	174.2 (3)
C3—C2—H2B	108.8		
			
C4—S1—C1—C2	−34.3 (3)	C5—N2—C4—N1	−179.3 (3)
S1—C1—C2—C3	59.4 (4)	C5—N2—C4—S1	3.5 (4)
C4—N1—C3—C2	25.2 (4)	C1—S1—C4—N1	7.7 (3)
C1—C2—C3—N1	−54.0 (4)	C1—S1—C4—N2	−175.3 (2)
C3—N1—C4—N2	178.6 (3)	C4—N2—C5—N3	−177 (3)
C3—N1—C4—S1	−4.3 (4)		

### Hydrogen-bond geometry (Å, °)

**Table:**

*D*—H···*A*	*D*—H	H···*A*	*D*···*A*	*D*—H···*A*
N1—H1A···N3^i^	0.86	2.12	2.926 (4)	156
C3—H3B···S1^ii^	0.99	2.74	3.468 (3)	131

Symmetry codes: (i) −*x*+1, *y*+1/2, −*z*+1/2; (ii) −*x*+2, *y*+1/2, −*z*+3/2.
